# A New Hyaluronan Modified with β-Cyclodextrin on Hydroxymethyl Groups Forms a Dynamic Supramolecular Network

**DOI:** 10.3390/molecules24213849

**Published:** 2019-10-25

**Authors:** Jelica Kovačević, Zdeňka Prucková, Tomáš Pospíšil, Věra Kašpárková, Michal Rouchal, Robert Vícha

**Affiliations:** 1 Department of Chemistry, Faculty of Technology, Tomas Bata University in Zlín, Vavrečkova 275, 760 01 Zlin, Czech Republic; jelica.kovacevic1@gmail.com (J.K.); pruckova@utb.cz (Z.P.); rouchal@utb.cz (M.R.); 2 Centre of the Region Haná for Biotechnological and Agricultural Research, Department of Chemical Biology and Genetics, Faculty of Science, Palacký University Olomouc, Šlechtitelů 241/27, CZ-783 71 Olomouc, Czech Republic; tomas.pospisil@upol.cz; 3 Department of Fat, Surfactant and Cosmetics Technology, Faculty of Technology, Tomas Bata University in Zlín, Vavrečkova 275, 760 01 Zlin, Czech Republic; vkasparkova@utb.cz

**Keywords:** cyclodextrin, sodium hyaluronan, click reaction, host-guest systems, supramolecular network

## Abstract

A new hyaluronan derivative modified with β-cyclodextrin units (CD-HA) was prepared via the click reaction between propargylated hyaluronan and monoazido-cyclodextrin (CD) to achieve a degree of substitution of 4%. The modified hyaluronan was characterized by ^1^H-nuclear magnetic resonance spectroscopy (NMR) and size exclusion chromatography. Subsequent ^1^H-NMR and isothermal calorimetric titration experiments revealed that the CD units on CD-HA can form virtual 1:1, 1:2, and 1:3 complexes with one-, two-, and three-site adamantane-based guests, respectively. These results imply that the CD-HA chains used the multitopic guests to form a supramolecular cross-linked network. The free CD-HA polymer was readily restored by the addition of a competing macrocycle, which entrapped the cross-linking guests. Thus, we demonstrated that the new CD-HA polymer is a promising component for the construction of chemical stimuli-responsive supramolecular architectures.

## 1. Introduction

Biopolymers are defined as polymers that are produced under natural conditions, from natural sources, or by chemical synthesis from biological materials. Biopolymers are synthesized within cells by enzymatic processes [1]. Biopolymers from the glycosaminoglycan series, namely, hyaluronic acid (HA), are of particular interest since they are water soluble, biodegradable, biocompatible, and abundant in nature. Biopharmaceutical properties, such as biocompatibility, biodegradability, and absence of immunotoxicity and cytotoxicity, make biopolymers ideal candidates for use in drug delivery [2,3], as drug carriers to prolong the effects of a drug [4,5], or for use as bioresorbable scaffolds in tissue engineering [6,7].

Natural polymers can be chemically modified to introduce different functional groups in the polymer chain, improve the properties of the polymer, and enable new functions. Studies on the chemical modifications of HA have been mainly concerned with cross-linking and grafting. The chemical structure of HA provides the three most commonly used sites for covalent modification, i.e., carboxylate groups, hydroxyl groups, and -NHCOCH_3_ groups [8]. Hydroxyl groups are usually cross-linked via an ether linkage, and carboxylic acid groups are cross-linked via an ester linkage. The chemical modification of the -NHCOCH_3_ group includes deacetylation, amidation, and hemiacetylation. Amidation methods are used for the deacetylation of the *N*-acetyl groups, and these reactions are usually performed using hydrazine sulphate [9,10,11]. The most common modification of HA is cross-linking, which leads to the preparation of HA-based hydrogels [12,13]. Hyaluronic acid can be cross-linked by bisepoxide [14], divinylsulphone derivatives under alkaline conditions [15], or glutaraldehyde [16], 1-ethyl-3-(3-dimethylaminopropyl) carbodiimide hydrochloride [16], and biscarbodiimide [17] under acidic conditions. In these cases, covalent bonds are used for the cross-linking of the polymer chains, and, therefore, any reversible modification of the polymer properties is disabled. In contrast, when the supramolecular host-guest approach is employed, the degree of cross-linkage can be controlled due to the reversible nature of supramolecular interactions [18].

Cyclodextrins (CDs), i.e., natural macrocyclic oligosaccharides with six to eight d-glucose units, have been studied in host-guest chemistry for the construction of supramolecular aggregates due to their hydrophobic cavities. Supramolecular polymers employing β-CD (a macrocycle consisting of seven glucose units) as a host have been constructed to improve stimuli-responsive properties [19]. Cyclodextrins can be easily modified on their narrow rim, which is decorated by primary hydroxyl groups. There are three strategies for constructing β-CD-based supramolecular polymers, such as using β-CD-based host-guest monomers to assemble a polymer chain [20], incorporating β-CD macrocycles in the side chain of the polymer [21] or assembling with a guest polymer and utilizing β-CD as building blocks with extensible sites to construct supramolecular polymers [22]. The grafting of β-CD to HA has been achieved by covalent approaches, including the conjugation of amine-modified β-CD to the carboxylic acid group of HA [23,24], the interaction of boric acid grafted to HA with the hydroxyl groups of β-CD [25], or by supramolecular assembly [26,27].

**Figure 1:**
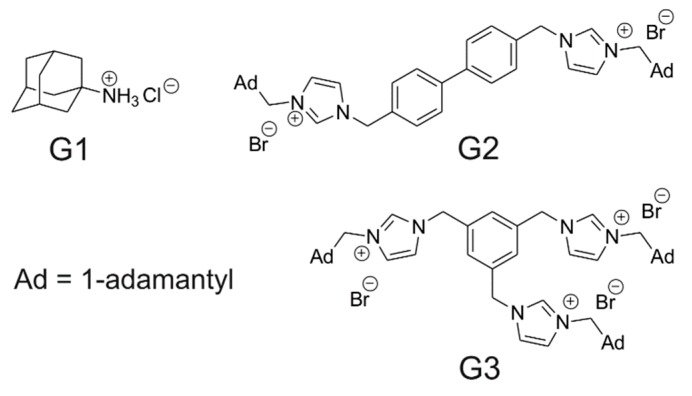
The adamantane-based supramolecular guests used in this study.

To the best of our knowledge, only one study has thus far been reported that describes the preparation and properties of HA modified with β-CD via a triazole linkage [28]. However, in this case, the cyclodextrin units were grafted onto carboxylate functional groups, which are believed to play an important role in the recognition of HA by cells [29,30]. The aim of this work was to examine whether the natural hyaluronan, which was conveniently modified with β-cyclodextrin units via the primary hydroxyl groups of HA, can form supramolecular networks with two- and three-site adamantane-based guest motifs (for structures, see Figure 1) in reversible manner.

## 2. Results and Discussion

### 2.1. Chemistry

**Scheme 1:**
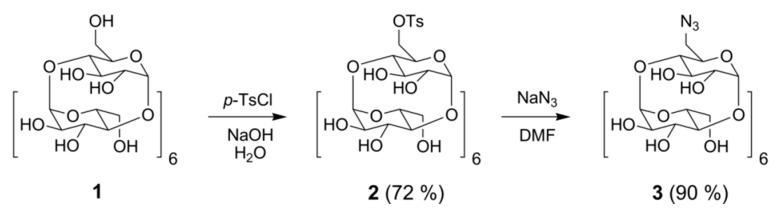
Monosubstitution of β-cyclodextrin (CD) intermediates at the primary hydroxyl group.

**Scheme 2:**
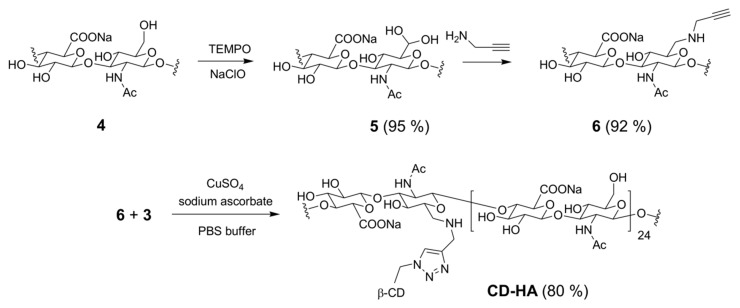
Synthesis of the cyclodextrin-modified hyaluronic acid (CD-HA) via a click reaction.

Initially, mono-6-(p-toluenesulphonyl)-β-cyclodextrin (**2**) was prepared from natural β-CD (**1**), where selective monotosylation was performed in the C6-OH position (Scheme 1). The reaction was performed in an alkaline aqueous solution according to a published procedure [31]. Covalent linkage between the tosyl group and β-CD was indicated by DOSY spectrum as shown in Figure S1. Afterwards, tosylated β-cyclodextrin (**2**) was readily converted to monoazido-CD (**3**) by an azidation reaction [32]. The azido group located on the primary rim of the CD scaffold is a suitable anchoring point for the introduction of an alkyne into the structure. Thus, a new CD-HA polymer was synthesized that combined the chemical modification of the HA chain with terminal alkyne groups via the sequence of partial selective oxidation, reductive amination, and final 1,3-dipolar cycloaddition between monoazido-β-CD (**3**) and propargylated HA (**6**) (Scheme 2).

Natural hyaluronan (**4**) was oxidized with 2,2,6,6-tetramethyl-4-acetamidopiperidine-1-oxyl (4-acetamide-TEMPO) [33] using sodium hypochlorite as a primary oxidant to produce aldehyde moieties (hydrated form of product (**5**) is shown in Scheme 2). Oxidation occurred at the C6-OH position, and the oxidized HA was grafted with primary amines carrying alkyne groups via Schiff imine intermediate mediation to produce modified hyaluronan (**6**). A new CD-HA polymer was prepared via the 1,3-dipolar cycloaddition between monoazido-CD and the propargyl derivative of HA. The coupling between monoazido-CD and the alkyne-terminated derivative of HA was successfully performed in 1 mM PBS at ambient temperature (Scheme 2).

### 2.2. Characterization of Products

The chemical structures of the monosubstituted CD intermediates were assessed using ^1^H-NMR, ESI-MS, and DOSY analyses (see the Supporting information and Materials and Methods section). The propargylated HA was characterized by means of ^1^H-NMR. The ^1^H-NMR spectrum shows signals for the *N*-acetyl group at 2.02 ppm. Signals of the HA skeletal H-atoms can be seen at 3.40–4.00 ppm. The remaining detected signals at 3.10 and 2.85 ppm can be assigned to the methylene protons at position C6. These signals confirmed the successful formation of the covalent linkage between propargylamine and HA. The ^1^H-NMR spectrum of the new CD-HA polymer showed a peak at 5.06 ppm, which is attributed to the H1 (β-CD). The substitution degree of 4% was calculated from the integration of the peak of the *N*-acetyl group at 2.00 ppm and the peak at 5.06 ppm, which is related to the anomeric proton of β-cyclodextrin (for the ^1^H-NMR spectrum, see Figure S3). It can be clearly seen in the DOSY spectrum (Figure S2) that the signals of the anomeric H-atoms from the CD macrocycle, anomeric H-atoms from the HA chain, skeletal H-atoms, and CH_3_ groups from the acetamido substituent lie on one line, i.e., display the same diffusion coefficient. This observation implies that the CD units are covalently linked to the HA polymer. Additionally, size exclusion chromatography was used to determine whether the HA polymer chain was markedly cleaved during the chemical transformations. As is shown in Figure S4, the peaks related to the original HA polymer, oxidized HA, propargylated HA, and CD-HA appeared at essentially the same retention time. Therefore, it can be inferred that no significant scission of the polymer backbone occurred.

### 2.3. Supramolecular Study of CD-HA

**Figure 2:**
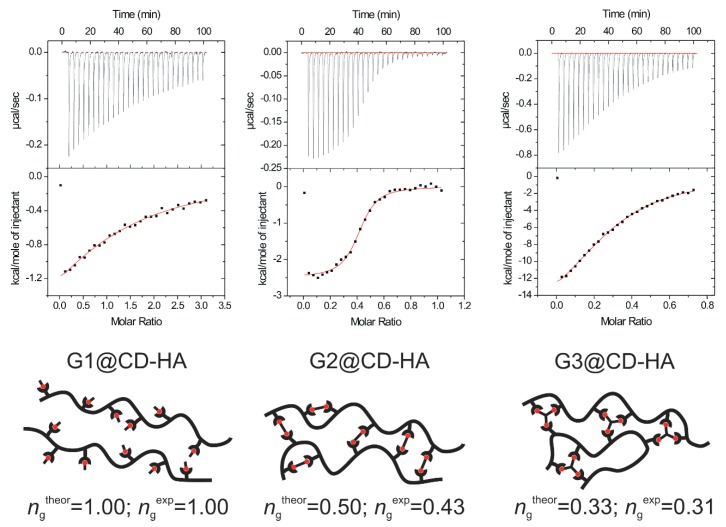
ITC results and interpretation of titrations of CD-HA with guests **G1**, **G2**, and **G3**.

We started our examination of the supramolecular properties of CD-HA by isothermal titration calorimetry (ITC) with the single-site guest **G1** (for structure, see Figure 1). Since the chemical nature of guest **G1** is well-defined and non-hygroscopic, it can be used as a standard to allow us to determine the actual concentration of available CD units. It was previously demonstrated that the guest **G1** forms a 1:1 complex with natural β-CD with a sufficiently high binding constant [34] to allow for the unambiguous analysis of titration data, namely, the stoichiometric parameter *n*, even at millimolar concentrations of the supramolecular components. Therefore, we performed an ITC titration of the CD-HA polymer with **G1** and calculated the actual concentration of CD units using the abovementioned parameter *n* from ITC and the degree of substitution, which was previously obtained by integrating the ^1^H-NMR spectrum. This concentration value was used as the input concentration of CD for the data processing of further titrations of CD-HA with **G2** and **G3**. Figure 2 clearly shows that relative to guest **G1**, an equivalency point was reached after the addition of one-half and one-third of the molar quantities of **G2** and **G3**, respectively (see Table 1 for ITC data). This means that, for instance, ditopic guest **G2** employs both its binding sites to saturate the CD units of CD-HA to form a supramolecular network, as depicted in Figure 2.

**Figure 3:**
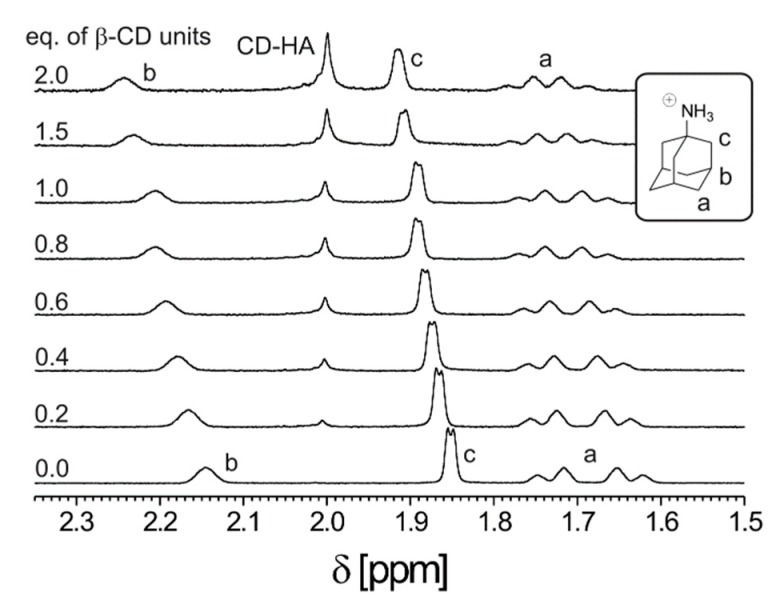
Stacking plot of portions of the ^1^H-NMR spectra recorded within the titration of **G1** with CD-HA (D_2_O, 30 °C).

Complementary information regarding the nature of the interactions between the supramolecular components was obtained using ^1^H-NMR titration. The stacked spectra that were recorded during the titration of CD-HA with the guest **G1** are displayed in Figure 3. Note that the signals of all hydrogen atoms, which can be assigned to the adamantane cage, are significantly shifted downfield. This phenomenon is well recognized and attributed to the deshielding effect of the cyclodextrin interior cavity [38]. In addition, complexation-induced shifts of the guest signals are very similar to those observed within the titration of **G1** with natural β-CD. Similar results were obtained within the ^1^H-NMR titration of **G2** and **G3**, respectively (see Figures S5 and S6). Therefore, we can conclude that the adamantane cage is included inside the CD cavity in all examined cases, and the nature of the interaction detected by ITC can be attributed to the formation of the supramolecular complex in a host-guest manner.

**Figure 4:**
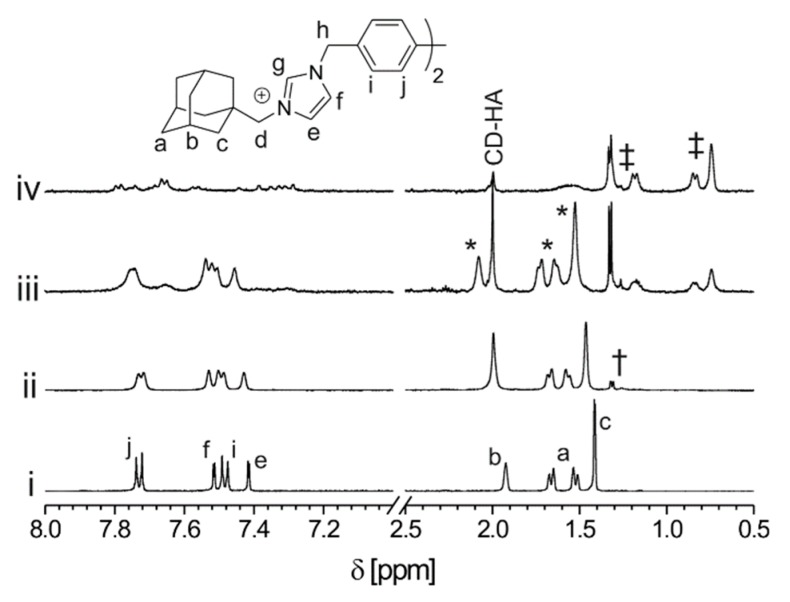
Stacking plot of portions of the ^1^H-NMR spectra recorded within the competitive experiment in D_2_O at 30 °C. Free guest **G2** (**i**); **G2** and 1 eq. of CD-HA (**ii**); **G2**, 1 eq. of CD-HA and 1 eq. of CB7 (**iii**); **G2**, 1 eq. of CD-HA and 2 eq. of CB7 (**iv**). The signal of the residual propan-2-ol is marked with † and signals of the adamantane cage complexed with β-CD and CB7 macrocycle are marked with * and ‡, respectively.

Afterwards, we performed a competitive experiment to determine whether the cross-linking agents **G2** or **G3** can be withdrawn from the CD-HA complex. The ^1^H-NMR spectra, which were recorded during this experiment, are shown in Figure 4. Initially, we added an equimolar quantity of CD-HA to the solution of guest **G2** to form a supramolecular network, as described above (lines i and ii in Figure 4). Subsequently, two molar equivalents of cucurbit[7]uril (CB7), with respect to **G2**, were added in two portions (lines iii and iv in Figure 4). Since cucurbit[7]uril is a well-known strong binding agent for the cationic derivatives of cage hydrocarbons [39,40], we expected that a complex of **G2** with CB7 would be preferred in this mixture. After the first addition of CB7, a new set of **G2** signals appeared. Since the new signals related to the adamantane cage were significantly shifted upfield (marked with ‡ in Figure 4), we infer that the complex of **G2**@CB7 formed. Note that the remaining signals of the adamantane cage are markedly shifted downfield (asterisked in Figure 4, line iii). This can be explained by a change in the environment, as the supramolecular network is partially cleaved. After the second addition of CB7, the signals related to the adamantane cage complexed inside the β-CD units completely disappeared. It should be noted that complex **G2**@CB7 was sparingly soluble in this system, and a colorless precipitate was formed. Since we obtained similar results with the guest **G3**, we infer that our new hyaluronan derivative is capable of forming supramolecular networks that can be modulated by a competitive approach.

## 3. Materials and Methods

### 3.1. General

Sodium hyaluronate (HA, Mn = 67.26 × 10^3^ g·mol^−1^, Mw = 174.27 × 10^3^ g·mol^−1^) was provided by Contipro Pharma (Dolní Dobrouč, Czech Republic) and characterized prior to use by size exclusion chromatography (Breeze 1525, Waters, Milford, MA US; column Shodex OH pack SB-806 HQ, 30 cm; detector RI; calibrated to pullulan 0.18–642 kg·mol^−1^). All other chemicals were purchased from Merck (Darmstadt, Germany). The purification of the modified HA was performed using a dialysis tubing cellulose membrane (14 kg·mol^−1^ MWCO, avg. flat width 33 mm, Merck, Darmstadt, Germany) and SnakeSkin dialysis tubing (3.5 kg·mol^−1^ MWCO, avg. flat width 34 mm, Thermo Scientific, Waltham, MA, USA). Milli-Q (Merck, Darmstadt, Germany) water was used for preparation of modified HA. HA was purified by a Direct-Q3 UV purification system (Merck, Darmstadt, Germany). Guests **G2** [36] and **G3** [37] were prepared via a conventional quaternization reaction and were reported previously, while the guest **G1** was purchased from Merck.

^1^H-NMR and ^13^C-NMR experiments were performed on an Avance TM-III 500 instrument (Bruker BioSpin, Billerica, MA, US) operating at frequencies of 500.11 MHz (^1^H) and 125.77 MHz (^13^C). ^1^H-NMR and ^13^C-NMR chemical shifts were referenced to the signal of the solvent [^1^H: δ (residual HDO) = 4.70 ppm, δ (residual DMSO-*d*_5_) = 2.50 ppm]. The degree of substitution (DS) was determined from ^1^H-NMR spectra as the average number of substituted disaccharides units per 100 units. Diffusion-ordered spectra (DOSY) were collected using an ECA-500 spectrometer (Jeol, Tokyo, Japan) operating at a frequency of 500.16 MHz (^1^H) and a bpp-led-dosy-pfg pulse sequence. The DOSY acquisition parameters were as follows: diffusion time 0.1 s, linear array of gradient strength (from 20 to 300 mT·m^−1^, 16 points), acquisition time 2.61 s, relaxation delay 8 s, 4 dummy scans and 1024–2048 total scans. Data were processed using the DELTA software package (ver. 5.0.5.1, Jeol, Tokyo, Japan).

Fourier transform infrared spectra were recorded on an ALPHA FTIR spectrometer (Bruker Optik GmbH, Ettlingen, Germany). The samples were measured in KBr pellets using 16 scans, and absorption spectra were scanned within the range of 400–4000 cm^−1^.

Electrospray mass spectra were recorded using an amaZon X ion-trap mass spectrometer (Bruker Daltonik GmbH, Bremen, Germany) equipped with an electrospray ionization source. All experiments were conducted in both positive and negative ion polarity mode. Each sample (5 μg∙mL^−1^) was infused into the ESI source in a CH_3_OH:H_2_O (1:1, *v*:*v*) solutions using a syringe pump with a constant flow rate of 3 μL∙min^−1^. The instrumental conditions used for the measurements are as follows: electrospray voltage of ± 4.2 kV, capillary exit voltage of ±140 V, drying gas temperature of 220 °C, drying gas flow rate of 6.0 dm^3^·min^−1^, and nebulizer pressure of 55.16 kPa.

Size exclusion chromatography (Shimadzu Prominence UFLC, Kyoto, Japan) with a refractive index detector was used to characterize the modified polymers. Samples of HA (5 mg∙mL^−1^) were prepared in 0.2 m NaNO_3_ + 0.01 m NaH_2_PO_4_, pH = 7 buffer. Measurements were performed at 40 °C using PL aquagel-OH 60 and 40 (2 × 30 cm) columns (Agilent, Santa Clara, CA, USA) with a flow rate of 0.8 mL∙min^−1^.

The association constants and thermodynamic parameters for the complexation of the guests (**G1**, **G2**, **G3**) with CD-HA were determined using a VP-ITC instrument (MicroCal, US). Isothermal titration calorimetry measurements were carried out in a 0.1 m NaNO_3_, 0.005 m NaH_2_PO_4_ buffer at pH = 5.03. The CD-HA solution (0.62 mg∙cm^−3^) was placed in the sample cell where the guest solution was added in a series of 29 injections (10 μL per injection). The concentrations of the **G1**, **G2**, and **G3** were 0.50 mM, 0.25 mM, and 0.18 mM, respectively. The raw experimental data were analyzed with MicroCal ORIGIN software (ver. 7.0, OriginLab, Northampton, MA, USA).

### 3.2. Preparation of 6-O-Monotosyl-6-Deoxy-β-Cyclodextrin (**2**)

Compound **2** was prepared by the reaction of β-CD with p-toluenesulphonyl chloride (TsCl) in alkaline aqueous medium according to the published procedure [31], with minor modifications. β-Cyclodextrin (1.00 g, 0.88 mmol) was dispersed in an aqueous 0.4 M NaOH solution. Afterwards, p-TsCl (0.71 g, 3.72 mmol) was slowly added in portions, and the solution was vigorously stirred at 0 °C. After 2 h, the remaining solid TsCl was removed by suction. Hydrochloric acid (0.8 M) was added dropwise to the filtrate to adjust pH to 8, and a white precipitate appeared. The mixture was stored in a refrigerator at 4 °C overnight, and the precipitate was collected by filtration through a sintered glass funnel. The colorless solid was washed with ice-cold water and dried under vacuum (6 torr) at 60 °C to yield 0.81 g (72%) of monotosylated cyclodextrin. M.p. = 168–170 °C.

^1^H-NMR (DMSO-*d*_6_, δ ppm): 7.75 (d, *J* = 8.7 Hz, 2H, Ph), 7.43 (d, *J* = 8.2 Hz, 2H, Ph), 5.62–5.86 (m, 14H, OH2, OH3), 4.85 (s, 3H, H1), 4.77 (s, 2H, H1), 4.42–4.48 (m, 5H, OH6), 4.21–4.24 (m, 1H, OH6), 3.46–3.66 (m, 30H, H1, H3, H5, H6), 3.17–3.38 (m, 14H, H2, H4 overlapped with H_2_O), 2.44 (s, 3H, -CH_3_). ESI-MS (pos.) *m*/*z* (%): 656.1 [M + H + Na]^2+^ (29), 664.1 [M + H + K]^2+^ (100), 1311.3 [M + Na]^+^ (15). ESI-MS (neg.) *m*/*z* (%): 643.1 [M − 2H]^2−^ (100), 1287.3 [M − H]^−^ (57). FTIR (KBr, cm^−1^) 3385 (O-H), 2928 (C-H), 1643 (C=C), 1030 (C-O).

### 3.3. Preparation of 6-O-Monoazido-6-Deoxy-β-Cyclodextrin (**3**)

Compound **2** (0.50 g, 0.38 mmol) was suspended in dimethylformamide (DMF), and the mixture was heated at 80 °C for 30 min [32]. Subsequently, NaN_3_ (0.12 g, 1.84 mmol) was slowly added to the solution, and the reaction mixture was stirred for 24 h at 80 °C. Afterwards, the crude product was precipitated by the addition of acetone. The colorless precipitate was collected by suction and dried in vacuum at 60 °C to yield 0.40 g (90%) of monoazidocyclodextrin. M.p. = 208–210 °C.

^1^H-NMR (DMSO-*d*_6_, δ ppm): 5.58–5.85 (m, 14H, OH2, OH3), 4.87 (m, 1H, H1), 4.83 (m, 6H, H1), 4.40–4.57 (m, 6H, OH6), 3.49–3.73 (m, 28H, H3, H5, H6), 3.30 (s, 14H, H2, H4 overlapped with H_2_O). ESI-MS (pos.) *m*/*z* (%): 591.7 [M + H + Na]^2+^ (23), 599.6 [M + H + K]^2+^ (100), 1182.4 [M + Na]^+^ (24). ESI-MS (neg.) *m*/*z* (%): 578.6 [M − 2H]^2−^ (79), 1158.3 [M − H]^−^ (100). FTIR (KBr, cm^−1^) 3383 (O-H), 2922 (C-H), 2110 (N_3_), 1157 (C-N).

### 3.4. Preparation of Oxidized Hyaluronan (**5**)

The selective oxidation of hyaluronic acid (**4**) was performed according to a slightly improved procedure, which has been published previously [33]. Hyaluronic acid sodium salt (1.00 g, 2.50 mmol) was dissolved in deionized water (50 mL). Subsequently, NaBr (0.13 g, 1.25 mmol) and Na_2_HPO_4_·12H_2_O (1.943 g, 5.43 mmol) were added. The reaction mixture was cooled in an ice water bath to 0 °C and stirred for 1 h. Finally, 4-acetamido-TEMPO (5.30 mg, 0.02 mmol) and 340 μL of 11% NaOCl were added to the reaction mixture under nitrogen atmosphere. The reaction was carried out for 15 min and quenched by the slow addition of 5% aqueous Na_2_S_2_O_3_. Afterwards, the solution was dialyzed against a 0.5% solution of salts (NaCl, NaHCO_3_). Finally, the product was precipitated with ethanol. Yield = 0.93 g (95%).

^1^H-NMR (HDO, δ ppm): 5.25 (s, 1H, CHO), 4.5 (m, 2H), 3.4–4.0 (m, 10H, skeletal H-atoms of HA), 2.0 (s, 3H, CH_3_). FTIR (KBr, cm^−1^) 3419 (C-OH), 2923, 1656, 1614 (-HC=O), 1413 (-O-C=O), 1153, 1080 (C-OH), 1039, 607.

### 3.5. Preparation of Propargylated Hyaluronan (**6**)

Reductive amination of hyaluronic acid was performed according to a previously published procedure [33] with minor modifications. The oxidized HA (0.20 g, 0.50 mmol) was dissolved in deionized water. Afterwards, propargylamine hydrochloride (6.90 mg, 0.07 mmol) was slowly added, and the reaction mixture was stirred for 5 h. Subsequently, picoline borane complex (8.00 mg, 0.07 mmol) was added, and the reaction was carried out overnight at room temperature. The prepared solution was dialyzed in a 0.5% solution of NaCl and NaHCO_3_. The product was isolated by precipitation with ethanol. Yield = 0.18 g (92%).

^1^H-NMR (D_2_O, δ ppm): 4.5 (m, 2H), 3.4–4.0 (m, 10H), 3.1 (m, CH_2_), 2.85 (m, CH_2_), 2.0 (s, CH_3_). FTIR (KBr, cm^−1^) 3379 (C-OH), 2894, 2131 (C≡C), 1614, 1407, 1078, 613.

### 3.6. Conjugation of β-CD to HA

Hyaluronan polymer modified with β-cyclodextrin (CD-HA) was obtained via a click reaction between monoazido-CD **3** and propargylated hyaluronan **6**. Compound **3** (0.10 g, 0.08 mmol) was dissolved in 2 mL of 1 mmol phosphate buffer solution (PBS) at pH = 7.4 followed by the subsequent addition of propargylated HA (7.72 mg, 0.01 mmol). Furthermore, CuSO_4_·5H_2_O (2.15 mg, 0.008 mmol) and sodium ascorbate (1.70 mg, 0.008 mmol) were dissolved in 120 μL of PBS and slowly added into the reaction mixture. The reaction mixture was stirred for a few minutes and was diluted with 1 mL of water. Afterwards, the solution was dialyzed against the 0.5 mmol EDTA in order to remove the copper catalyst. The product was recovered by precipitation with ethanol and dried under vacuum to yield 0.14 g (80%) of colorless powder.

^1^H-NMR (D_2_O, δ ppm): 5.0 (s), 4.6 (d), 4.5 (d), 3.3–4.0 (m, skeletal H-atoms of HA), 2.0 (s, NHCOCH_3_). FTIR (KBr, cm^−1^) 3394 (N-H), 3028 (C-H), 2785 (C-H), 1673 (C=O), 1627 (C=O), 1396 (C-O), 1032, 710, 550.

## 4. Conclusions

In conclusion, a new hyaluronan polymer modified with β-cyclodextrin units at the C6 position of the *N*-acetylglucosamine ring was prepared via 1,3-dipolar cycloaddition between propargylated HA and monoazido-β-CD to reach a degree of substitution of 4%. Subsequent NMR and ITC experiments revealed that β-CD units on the CD-HA polymer form 1:1, 1:2, and 1:3 supramolecular aggregates in a host-guest manner with adamantane-based mono-, di-, and tritopic guests, respectively. Finally, we demonstrated that the supramolecular network can be readily cleaved to restore the original CD-HA by the addition of a suitable competitor for the multitopic guests. These results confirm our initial expectation that the new CD-HA polymer is a promising component for the construction of chemical stimuli-responsive supramolecular architectures.

## Figures and Tables

**Table 1:** Thermodynamic data for the guests **G1**–**G3**.

Guest	Host	*n* ^1^	*K*^2^ [dm^3^·mol^−1^]	−Δ*H* [kJ·mol^−1^]	Δ*S* [J·mol^−1^·K^−1^]
**G1**	β-CD	0.96	7.05 × 10^3^	24.5	−7
	CB7 ^3^	ng ^4^	1.7 × 10^14^	80.6	0
	CD-HA	1	1.12 × 10^4^	13.9	32
**G2**	β-CD ^5^	0.49	9.20 × 10^4^	59.0	−99
	CB7 ^5^	0.56	1.35 × 10^12^	ng	ng
	CD-HA	0.43	1.14 × 10^6^	11.4	79
**G3**	β-CD ^6^	0.37	1.02 × 10^5^	85.7	−187
	CB7 ^6^	0.39	4.84 × 10^10^	173.8	−369
	CD-HA	0.31	1.00 × 10^5^	87.6	−193

^1^*n* values for CD-HA are given relative to the *n* value for **G1**; ^2^ values of apparent *K* are given for CD-HA; ^3^ reference [35]; ^4^ not given, 1:1 stoichiometry was assumed; ^5^ reference [36]; ^6^ reference [37].

## Supplementary Materials

The following are available online; Figure S1: DOSY spectrum of the crude product of monotosylation of β-CD, Figure S2: DOSY spectrum of CD-HA, Figure S3: ^1^H-NMR spectrum of CD-HA, Figure S4: SEC results of original hyaluronan (HA, **4**), oxidized hyaluronan (OX-HA, **5**), propargylated hyaluronan (Prop-HA, **6**), and final hyaluronane modified by β-CD units (CD-HA), Figure S5: Stacking plot of portions of the ^1^H-NMR spectra recorded within the titration of **G2** with CD-HA (D_2_O, 30 °C), Figure S6: Stacking plot of portions of the ^1^H-NMR spectra recorded within the titration of **G3** with CD-HA (D_2_O, 30 °C).
