# Supplementary material for: A New Hyaluronan Modified with β-Cyclodextrin on Hydroxymethyl Groups Forms a Dynamic Supramolecular Network
Source: Molecules. 2019 Oct 25;24(21):3849. doi: 10.3390/molecules24213849 (PMC6864629; doi:10.3390/molecules24213849)
Supplement: Supplementary file 1 [file molecules-24-03849-s001.pdf]

A new hyaluronan modified with β-cyclodextrin on hydroxymethyl groups forms a dynamic supramolecular network

Jelica Kovačević,[a] Zdeňka Prucková,[a] Tomáš Pospišil,[b] Věra Kašpárková,[c] Michal Rouchal,[a] Robert Vícha[a*]

[a] *Department of Chemistry, Faculty of Technology, Tomas Bata University in Zlín, Vavrečkova 275, 760 01 Zlin, Czech Republic*
[b] *Department of Chemical Biology and Genetics, Centre of the Region Haná for Biotechnological and Agricultural Research, Faculty of Science, Palacký University Olomouc, Šlechtitelů 241/27, CZ-783 71 Olomouc, Czech Republic*
[c] *Department of Fat, Surfactant and Cosmetics Technology, Faculty of Technology, Tomas Bata University in Zlín, Vavrečkova 275, 760 01 Zlin, Czech Republic*

Supporting information

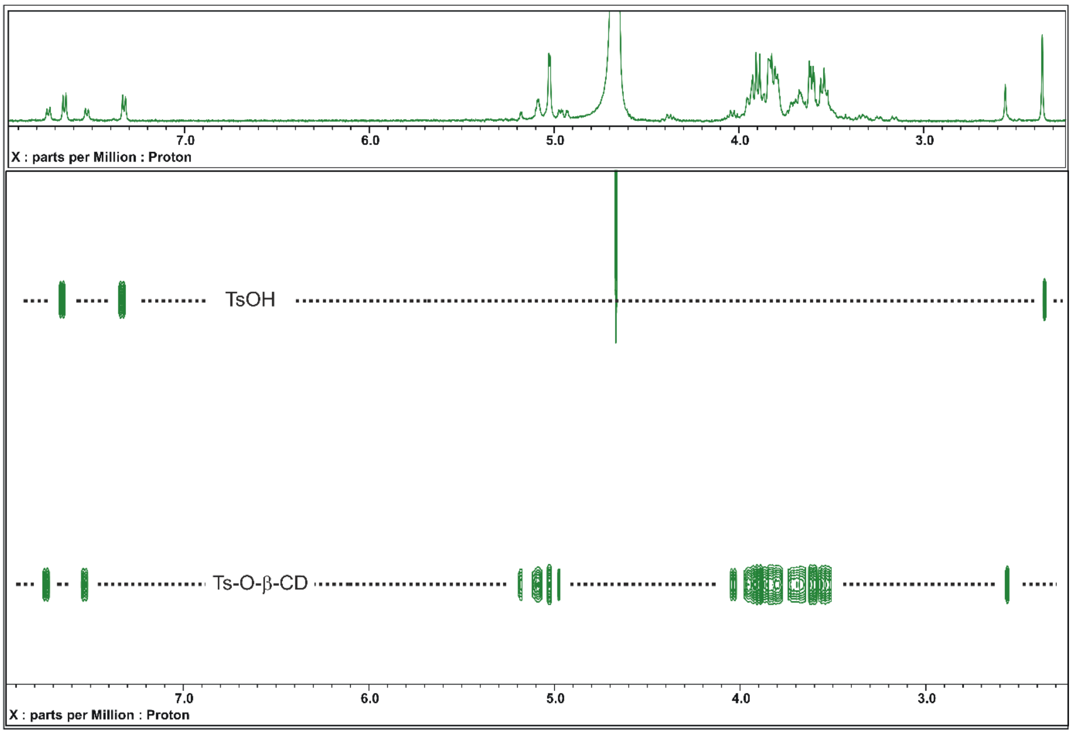

Figure S1 DOSY spectrum of the crude product of monotosylation of β-CD. Signals in upper line are related to the 4-methylbenzene sulphonic acid, signals in bottom line are related to the modified β-CD.

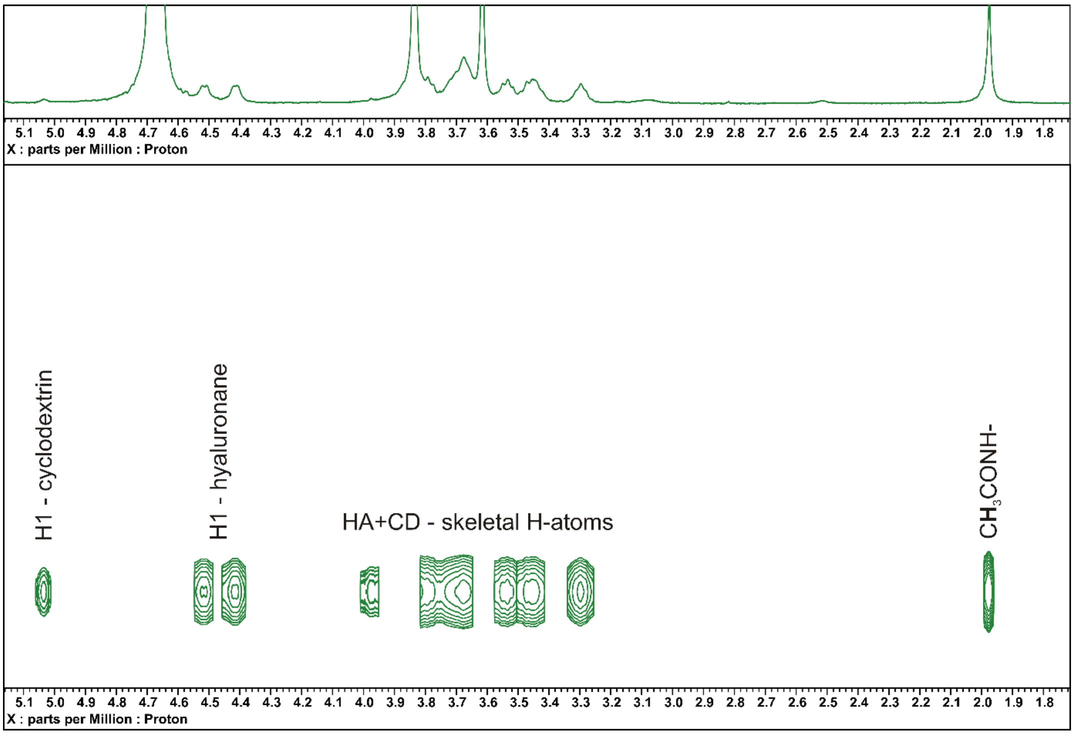

Figure S2 DOSY spectrum of hyaluronan modified by β-cyclodextrin units (CD-HA)

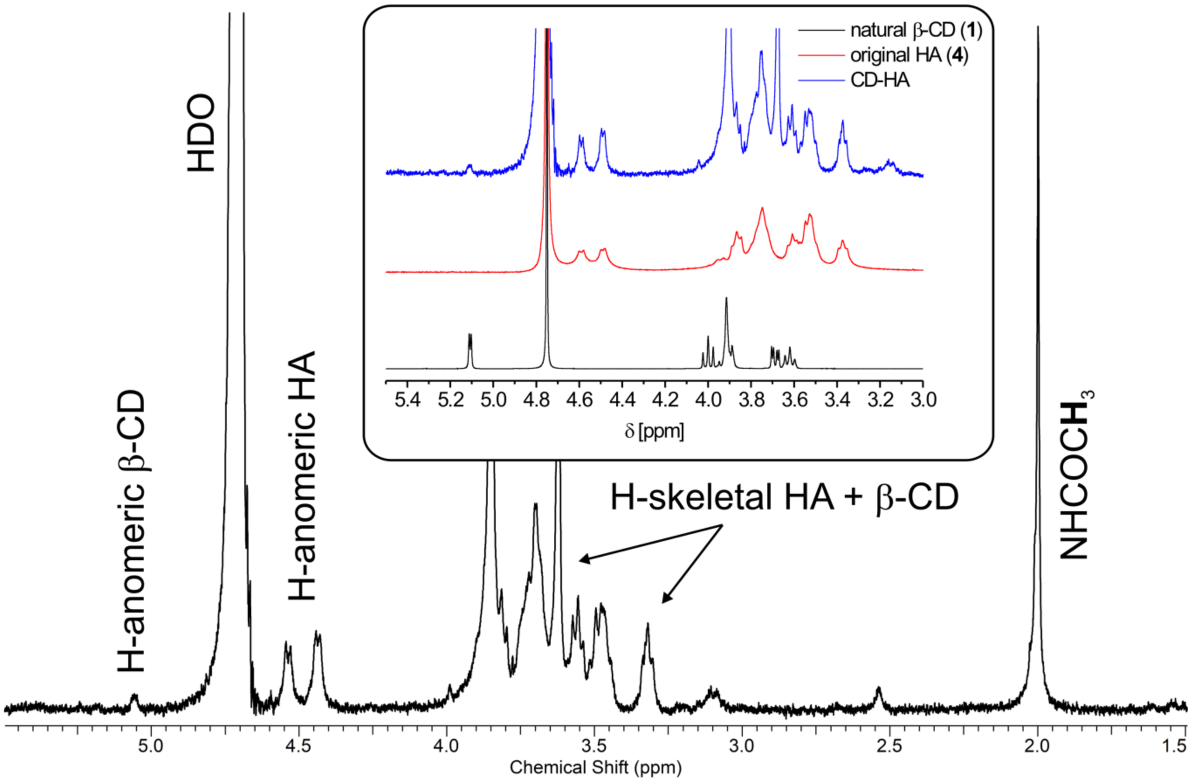

Figure S3 ^{1}H NMR spectrum of CD-HA. Comparison with the ^{1}H NMR spectra of the original HA and β-CD is inserted in the box.

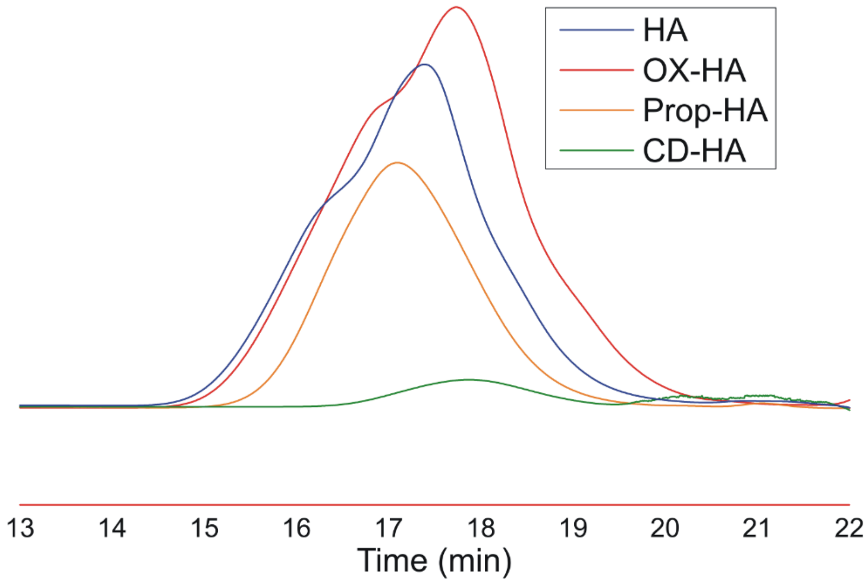

Figure S4 SEC results of original hyaluronan (HA, **4**), oxidized hyaluronan (OX-HA, **5**), propargylated hyaluronan (Prop-HA, **6**), and final hyaluronane modified by β-CD units (CD-HA).

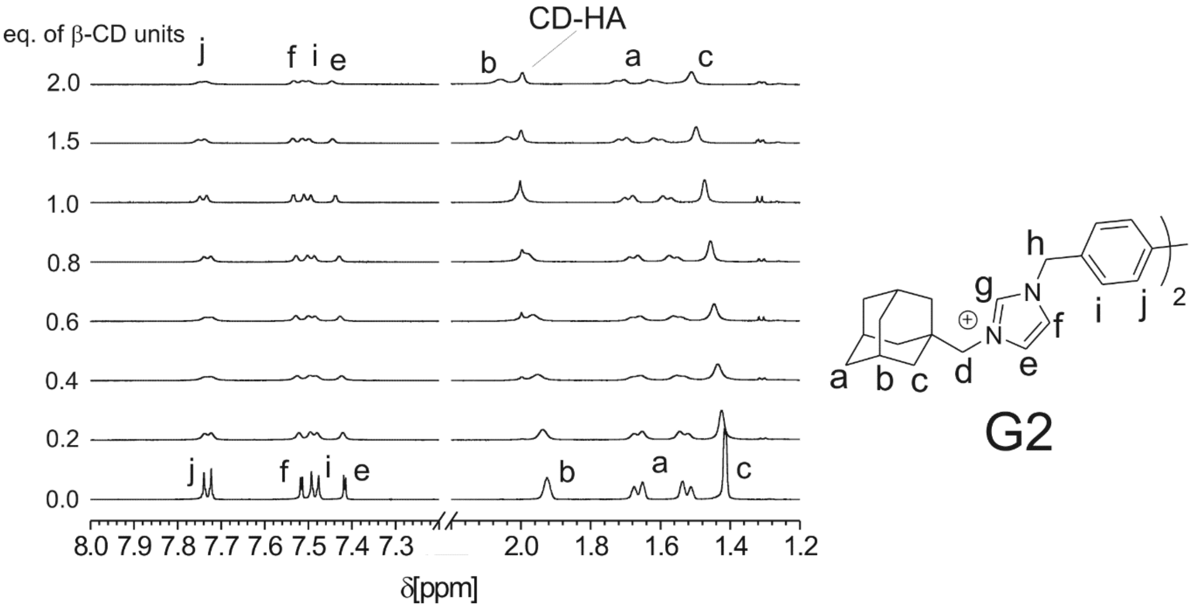

Figure S5 Stacking plot of portions of the ^{1}H NMR spectra recorded within the titration of G2 with CD-HA (D_2O, 30 °C).

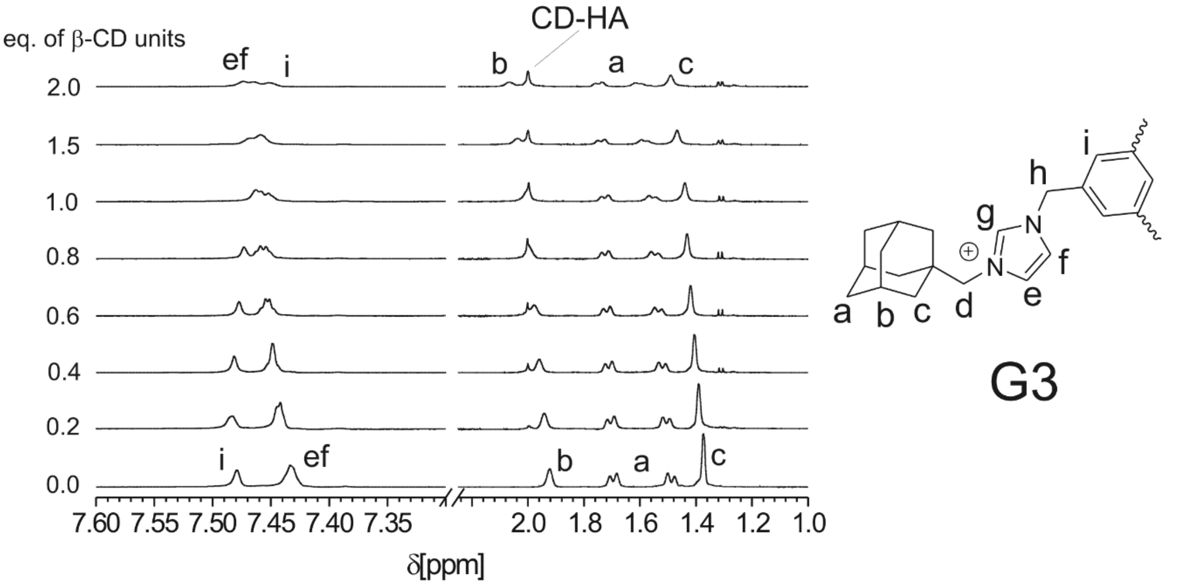

Figure S6 Stacking plot of portions of the ^{1}H NMR spectra recorded within the titration of G3 with CD-HA (D_2O, 30 °C).
